# Verification and dissection of one quantitative trait locus for grain size and weight on chromosome 1 in rice

**DOI:** 10.1038/s41598-021-97622-8

**Published:** 2021-09-14

**Authors:** Yi-chen Cheng, Guan Li, Man Yin, Tosin Victor Adegoke, Yi-feng Wang, Xiao-hong Tong, Jian Zhang, Jie-zheng Ying

**Affiliations:** 1 State Key Laboratory of Rice Biology and Chinese National Center for Rice Improvement, China National Rice Research Institute, Hangzhou, 310006 China; 2 State Key Laboratory of Subtropical Silviculture, Zhejiang A&F University, Lin’an, 311300 Zhejiang Province China

**Keywords:** Genetics, Plant sciences

## Abstract

Grain size and weight are the key traits determining rice quality and yield and are mainly controlled by quantitative trait loci (QTL). In this study, one minor QTL that was previously mapped in the marker interval of JD1009-JD1019 using the Huanghuazhan/Jizi1560 (HHZ/JZ1560) recombinant inbred line (RIL) population, *qTGW1-2*, was validated to regulate grain size and weight across four rice-growing seasons using twenty-one near isogenic line (NIL)-F_2_ populations. The twenty-one populations were in two types of genetic background that were derived from the same parents HHZ and JZ1560. Twelve F_9_, F_10_ or F_11_ NIL-F_2_ populations with the sequential residual heterozygous regions covering JD1009-RM6840 were developed from one residual heterozygote (RH) in the HHZ/JZ1560 RIL population, and the remaining nine BC_3_F_3_, BC_3_F_4_ or BC_3_F_5_ NIL-F_2_ populations with the sequential residual heterozygous regions covering JD1009-RM6840 were constructed through consecutive backcrosses to the recurrent parent HHZ followed with marker assistant selection in each generation. Based on the QTL analysis of these genetic populations, *qTGW1-2* was successfully confirmed to control grain length, width and weight and further dissected into two QTLs, *qTGW1-2a* and *qTGW1-2b*, which were respectively narrowed down to the marker intervals of JD1139-JD1127 (~ 978.2-kb) and JD1121-JD1102 (~ 54.8-kb). Furthermore, the two types of NIL-F_2_ populations were proved to be able to decrease the genetic background noise and increase the detection power of minor QTL. These results provided an important basis for further map-based cloning and molecular design breeding with the two QTLs in rice.

Rice (*Oryza sativa* L.) is one of the three major food crops, and feeds more than half of the consumers as their staple food in the world. Number of panicles per plant, number of grains per panicle, and grain weight are the three determined components of grain yield, among which grain weight is the direct factor to determine grain yield. Grain weight is generally evaluated by 1000-grain weight (TGW) and is closely related to grain length (GL), grain width (GW), grain thickness (GT) and length width ratio (LWR), which are comprehensively controlled by a large number of quantitative trait loci (QTL)^1,2^. GL and GW are stably inheritable traits that are less affected by environments, whereas GT and grain plumpness are easily affected by environmental factors and dependent on the filling process. Therefore, most QTLs for grain size and weight are map-based cloned through identifying GL, GW and TGW^3^.

In the past two decades, fine-mapping and cloning for yield traits, especially for grain weight, have achieved considerable progress. Up to now, 20 QTLs for grain weight and grain size have been cloned. Among them, both *GL7*^4^/ *GW7*^5^ and *GS9*^6^ have opposite allelic directions of additive effects on GL and GW, regulating grain size but hardly affecting grain weight. *GSA1*^7^ and *GW6a*^8^ have similar effects on GL and GW with same directions so that they have great influence on grain weight. The remaining 16 QTLs affect grain size and grain weight at the same time. Six of the 16 QTLs mainly control grain width and weight, including *GW2*^9^, *TGW2*^10^, *GS5*^11^, *qSW5*^12^/*GW5*^13^, *GW6*^14^ and *GW8*^15^, and the other ten QTLs mainly control grain length and weight, including *GS2*^16^/*GL2*^17^, *OsLG3*^18^, *qLGY3*^19^/*OsLG3b*^20^, *GS3*^2^, *GL3.1*^21^/*qGL3*^22^, *TGW3*^23^/*GL3.3*^24^, *GL4*^25^, *TGW6*^26^, *GL6*^27^ and *GLW7*^28^. Isolation and functional characterization of these QTLs have greatly enhanced our understanding of genetic control of grain size and weight in rice, but more investigations are needed to enrich the regulatory framework for these key agronomic traits^29^.

Although 20 QTLs with major effects have been successfully isolated, majority of these QTLs for grain size and weight have minor effects and are difficult to be repeatedly identified in different trials. Therefore, increasing the detection power of minor QTLs is the key for fine mapping or map-based cloning of them. For detecting minor QTLs, near isogenic line F_2_ (NIL-F_2_) populations are the ideal materials to eliminate the genetic noise of background and increase the detection power largely. In the NIL-F_2_ population, the region of target QTL is segregated with fixed genetic background, which will enable to eliminate the background noise and precisely estimate the effect of minor QTLs. NIL-F_2_ population can be produced by consecutive backcrossing strategy or residual heterozygous line method^30^. Consecutive backcrossing strategy includes continuous backcrosses to the recurrent parent and marker assistant selection in each generation. This type of NIL-F_2_ population has been used in cloning many QTLs^7,19,28,31^. NIL-F_2_ populations generated by self-pollinating residual heterozygotes (RH) in the recombinant inbred line (RIL) population have validated and dissected many QTLs for grain size and weight^32–34^.

In our previous study, we identified one stably expressed QTL regulating GL and grain weight, *qTGW1-2*/*qGL1-2*, using the HHZ/JZ1560 RIL population across two years^23^. To validate and fine map *qTGW1-2*/*qGL1-2*, two types of NIL-F_2_ populations including RH-derived population and advanced backcross population were constructed using HHZ and JZ1560 (Fig. 1). HHZ is an *indica* rice variety widely cultivated in China with small grains, and JZ1560 is a *japonica* material with super large grains.

**Figure 1:**
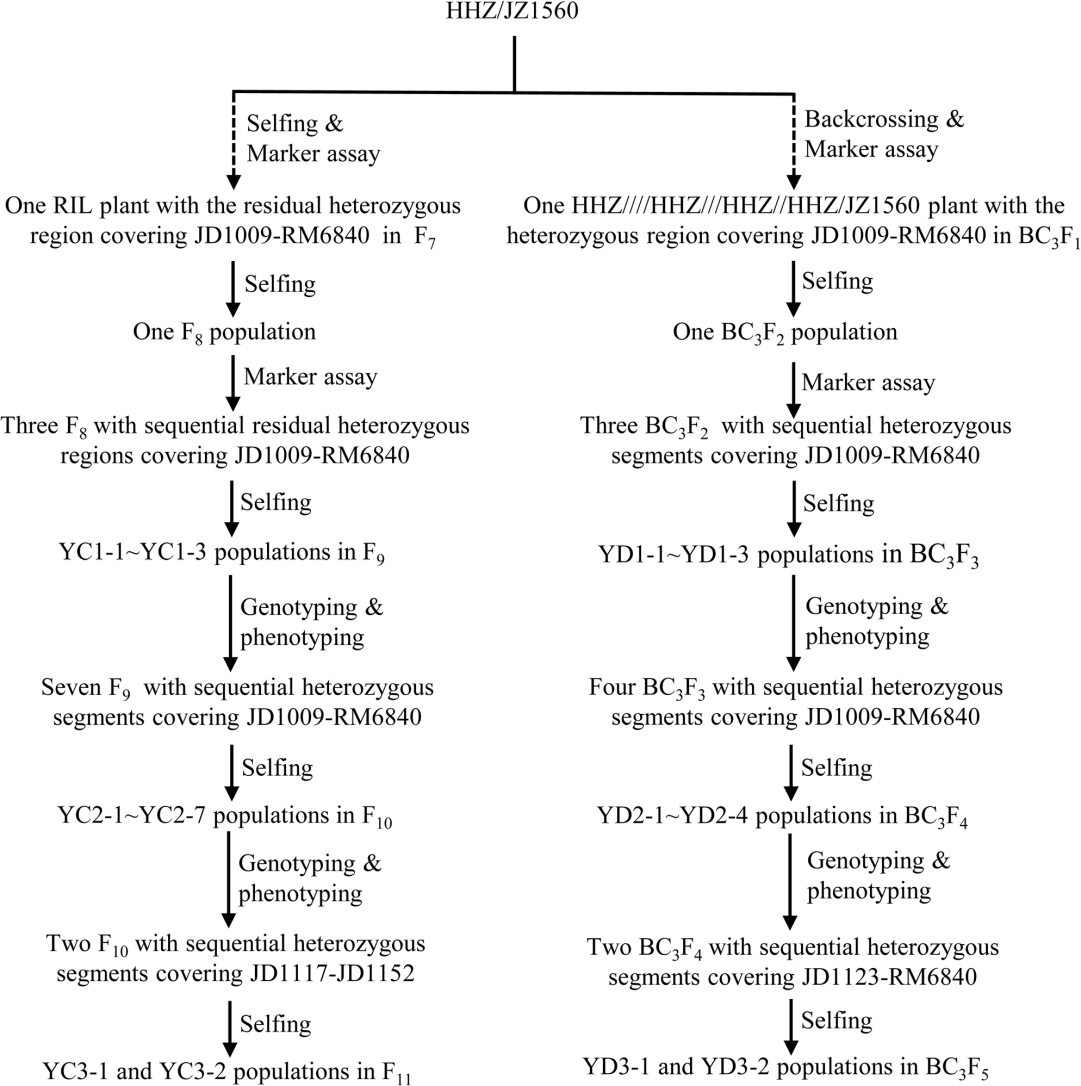
Development of two types of near isogenic line F_2_ populations.

## Results

### Validation and dissection of ***qTGW1-2*** using ten RH-derived NIL-F_2_ populations

Descriptive statistics of TGW, GL and GW in the ten RH-derived populations in F_9_ and F_10_ are shown in Table 1. Three traits in all the ten populations showed continuous distributions with low skewness and kurtosis, which suggested the typical phenotypic distributions of quantitative traits.

**Table 1:** Phenotypic performance of TGW, GL and GW in ten RH-derived populations in F_9_ or F_10._ TGW, 1000-grain weight (g); GL, grain length (mm); GW, grain width (mm).

Population	Trait	Mean	SD	CV	Range	Skew	Kurt
YC1-1	TGW	36.90	2.37	0.06	32.13–43.59	0.41	0.20
	GL	11.34	0.15	0.01	11.08–11.67	0.07	− 0.88
	GW	3.27	0.06	0.02	3.13–3.42	− 0.07	− 0.11
YC1-2	TGW	37.71	2.00	0.05	33.56–44.15	0.64	0.86
	GL	11.33	0.15	0.01	10.98–11.66	− 0.02	− 0.47
	GW	3.27	0.06	0.02	3.11–3.46	0.04	1.17
YC1-3	TGW	44.98	1.62	0.04	41.55–49.62	0.39	0.05
	GL	11.03	0.16	0.01	10.63–11.51	0.45	0.17
	GW	3.42	0.07	0.02	3.29–3.59	0.35	− 0.52
YC2-1	TGW	36.72	1.20	0.03	32.14–40.53	0.49	0.23
	GL	10.61	0.15	0.01	10.20–11.13	0.63	0.62
	GW	2.99	0.05	0.02	2.88–3.10	− 0.07	− 0.35
YC2-2	TGW	37.02	1.50	0.04	33.53–40.63	0.14	− 0.29
	GL	11.04	0.16	0.01	10.71–11.47	0.47	− 0.42
	GW	3.06	0.07	0.02	2.84–3.24	− 0.06	0.46
YC2-3	TGW	37.57	1.13	0.03	33.53–40.82	0.79	0.55
	GL	10.67	0.17	0.02	10.23–11.19	0.43	0.95
	GW	3.10	0.04	0.01	3.00–3.21	0.24	− 0.22
YC2-4	TGW	39.38	1.55	0.04	35.09–43.21	0.11	− 0.33
	GL	10.62	0.18	0.02	10.25–11.06	0.29	− 0.51
	GW	3.13	0.06	0.02	2.90–3.30	− 0.16	0.96
YC2-5	TGW	36.58	1.05	0.03	33.83–38.88	0.14	− 0.35
	GL	10.44	0.13	0.01	10.09–10.80	0.17	− 0.21
	GW	3.05	0.04	0.01	2.92–3.16	0.08	0.08
YC2-6	TGW	36.81	1.19	0.03	33.98–39.99	0.27	− 0.32
	GL	10.56	0.14	0.01	10.26–10.96	0.43	0.04
	GW	3.04	0.04	0.01	2.94–3.16	0.08	− 0.34
YC2-7	TGW	37.02	1.16	0.03	33.94–39.69	0.14	− 0.48
	GL	10.55	0.13	0.01	10.27–10.96	0.34	0.09
	GW	3.03	0.04	0.01	2.94–3.11	− 0.11	− 0.50

**Figure 2:**
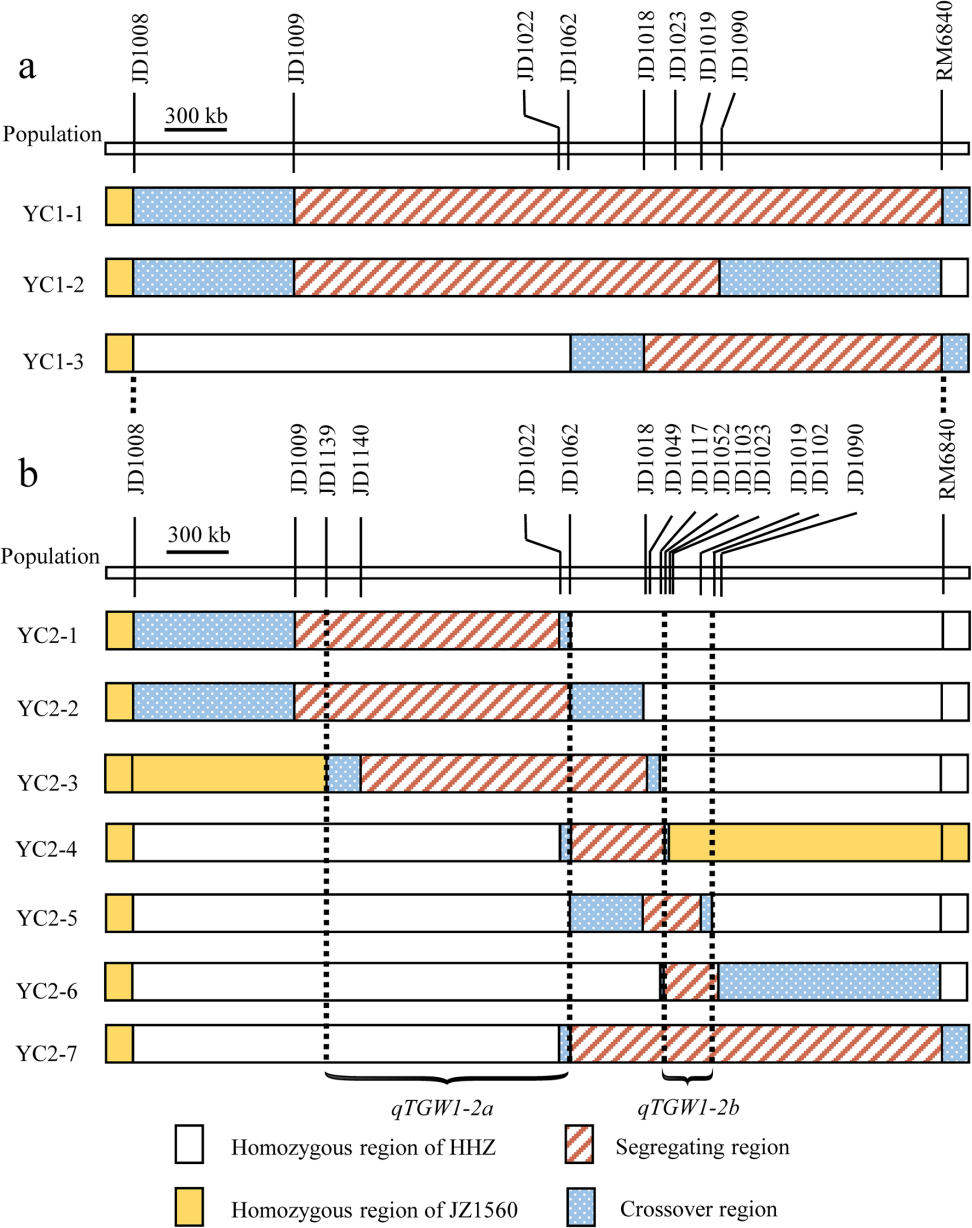
Genotypic compositions of the ten residual heterozygote-derived F_2_ populations in the segregating regions.

To confirm the location and genetic effect of *qTGW1-2*, three plants with sequential heterozygous regions covering JD1009-RM6840 were selected from one population in F_8_ and were self-crossed to generate three NIL-F_2_ populations named as YC1-1, YC1-2 and YC1-3 (Table 2; Fig. 1). Three segmental linkage maps were constructed for YC1-1, YC1-2 and YC1-3, respectively (Fig. 2a).

**Table 2:** Rice populations and field experiments. HZ, Hangzhou, Zhejiang Province; LS, Lingshui, Hainan Province.

Population			Segregating region	Number of plants	Location and growing season
Name	Generation				
**RH-derived populations**					
YC1-1		F_9_	JD1009-RM6840	120	HZ: May–September 2018
YC1-2		F_9_	JD1009-JD1090	128	HZ: May–September 2018
YC1-3		F_9_	JD1018-RM6840	224	HZ: May–September 2018
YC2-1		F_10_	JD1009-JD1022	190	HZ: May–September 2019
YC2-2		F_10_	JD1009-JD1062	190	HZ: May–September 2019
YC2-3		F_10_	JD1140-JD1049	104	HZ: May–September 2019
YC2-4		F_10_	JD1062-JD1052	190	HZ: May–September 2019
YC2-5		F_10_	JD1018-JD1019	190	HZ: May–September 2019
YC2-6		F_10_	JD1052-JD1090	190	HZ: May–September 2019
YC2-7		F_10_	JD1062-RM6840	190	HZ: May–September 2019
YC3-1		F_11_	JD1152-JD1136	190	HZ: May–September 2020
YC3-2		F_11_	JD1136-JD1121	190	HZ: May–September 2020
**Backcross populations**					
YD1-1		BC_3_F_3_	JD1009-JD1052	190	HZ: May–September 2019
YD1-2		BC_3_F_3_	JD1009-JD1090	190	HZ: May–September 2019
YD1-3		BC_3_F_3_	JD1018-RM6840	190	HZ: May–September 2019
YD2-1		BC_3_F_4_	JD1009-JD1133	190	LS: December 2019–April 2020
YD2-2		BC_3_F_4_	JD1127-JD1052	190	LS: December 2019–April 2020
YD2-3		BC_3_F_4_	JD1022-JD1090	190	LS: December 2019–April 2020
YD2-4		BC_3_F_4_	JD1134-RM6840	190	LS: December 2019–April 2020
YD3-1		BC_3_F_5_	JD1134-JD1159	190	HZ: May–September 2020
YD3-2		BC_3_F_5_	JD1152-RM6840	190	HZ: May–September 2020

Based on the genotype and phenotype data, *qTGW1-2* for TGW, GL and GW was identified in all the three populations, and the enhancing alleles for the three traits in all the three populations were all derived from JZ1560 (Table 1). In YC1-1, *qTGW1-2* showed the additive effects of 1.66 g, 0.11 mm and 0.06 mm for TGW, GL and GW, and explained 19.3%, 24.4% and 33.9% of phenotypic variations (*R*^2^). In YC1-2, the additive effects for TGW, GL and GW were 1.59 g, 0.11 mm and 0.04 mm, explaining 23.8%, 26.3% and 18.9% of phenotypic variations. Similar additive effects in the same direction and similar *R*^2^ values confirmed the existence of *qTGW1-2* in the common segregating region of YC1-1 and YC1-2. Compared with the additive effects for TGW in YC1-1 and YC1-2, a relatively smaller additive effect of 0.92 g was detected in the remaining population YC1-3. We further compared the segregating regions of JD1009-RM6840, JD1009-JD1090 and JD1018-RM6840 in YC1-1, YC1-2 and YC1-3 populations. The segregating regions of YC1-1 and YC1-2 covered all or part of the segregating region of YC1-3. These results suggested that there might be two or more QTLs with the same direction of additive effects in the primary interval of *qTGW1-2*.

To confirm our conjecture, the segregating region responsible for *qTGW1-2* should be subdivided into smaller heterozygous segments. Seven recombinant plants with sequential segregating regions covering the interval JD1009-RM6840 were selected from YC1-1, YC1-2 and YC1-3 populations and selfed to develop F_10_ RH-derived F_2_ populations, namely YC2-1, YC2-2, YC2-3, YC2-4, YC2-5, YC2-6 and YC2-7 (Table 2; Fig. 1). The seven F_10_ populations carried smaller segregating regions. TGW, GL and GW of each plant in the seven populations were measured and showed continuous segregations in each population (Table 1). Combined the genotype and phenotype information of each plant in the seven populations, seven segmental linkage maps were constructed (Fig. 2b). QTL analysis results for TGW, GL and GW using these populations are shown in Table 3. Except for YC2-4, QTLs responsible for TGW, GL and GW were detected in the remaining six populations. Since YC2-1 and YC2-2 showed the common segregating region (JD1139-JD1022) and no overlapping segregating region with YC2-5 and YC2-6, these results indicated that there should be two QTLs in the target interval of *qTGW1-2*. Furthermore, the segregating region of YC2-3 overlapped with the common segregating region (JD1139-JD1022), and similar additive effects for TGW, GL and GW were observed in YC1-1, YC1-2 and YC1-3. The result indicated that one QTL controlling TGW, GL and GW was located in the common segregating region of YC2-1, YC2-2 and YC2-3 flanked by JD1139 and JD1062, corresponding to a 1.2-Mb region in the Nipponbare genome. We designated this QTL as *qTGW1-2a*. Due to the fact that no QTL for all the three traits detected in YC2-4, the other QTL for TGW, GL and GW should be located in the common segregating region of YC2-5, YC2-6 and YC2-7 but outside the segregating region of YC2-4, with the interval flanked by JD1052 and JD1102, corresponding to a 235.1-kb region in the Nipponbare genome. We named this QTL as *qTGW1-2b*.

**Table 3:** QTLs detected for TGW, GL, and GW in ten RH-derived populations in F_9_ and F_10._ TGW, 1000-grain weight (g); GL, grain length (mm); GW, grain width (mm); *A*, additive effect of replacing a HHZ allele with a JZ1560 allele; *D*, dominance effect; *R*^2^, proportion of phenotypic variance explained by the QTL effect; ns, no significance.

Population	Segregating region	Trait	*LOD*	*A*	*D*	*R*^2^ (%)
YC1-1	JD1009-RM6840	TGW	5.18	1.66	− 0.77	19.3
		GL	7.07	0.11	0.04	24.4
		GW	9.80	0.06	− 0.01	33.9
YC1-2	JD1009-JD1090	TGW	6.78	1.59	− 0.38	23.8
		GL	7.98	0.11	− 0.04	26.3
		GW	5.68	0.04	− 0.02	18.9
YC1-3	JD1018-RM6840	TGW	8.34	0.92	0.03	17.0
		GL	12.47	0.11	− 0.02	24.4
		GW	3.70	0.03	0.00	7.9
YC2-1	JD1009-JD1022	TGW	6.71	0.67	− 0.01	15.4
		GL	8.24	0.10	− 0.03	18.5
		GW	7.20	0.03	0.00	16.4
YC2-2	JD1009-JD1062	TGW	6.78	0.85	0.00	15.8
		GL	4.13	0.07	0.01	9.9
		GW	5.28	0.04	0.01	12.5
YC2-3	JD1140-JD1049	TGW	4.81	0.57	− 0.66	19.7
		GL	3.57	0.08	− 0.09	15.0
		GW	3.33	0.03	− 0.01	14.1
YC2-4	JD1062-JD1052	TGW	ns	ns	ns	ns
		GL	ns	ns	ns	ns
		GW	ns	ns	ns	ns
YC2-5	JD1018-JD1019	TGW	6.36	0.59	0.09	14.9
		GL	3.45	0.06	0.00	8.4
		GW	3.01	0.02	0.00	7.4
YC2-6	JD1052-JD1090	TGW	13.11	0.87	− 0.01	28.2
		GL	9.39	0.09	0.01	21.2
		GW	7.66	0.03	0.00	17.6
YC2-7	JD1062-RM6840	TGW	14.70	0.97	− 0.06	32.1
		GL	12.51	0.11	− 0.03	27.4
		GW	10.49	0.03	0.00	23.8

### Validation and dissection of *qTGW1-2* using seven NIL-F_2_ populations in BC_3_F_3_ and BC_3_F_4_

**Figure 3:**
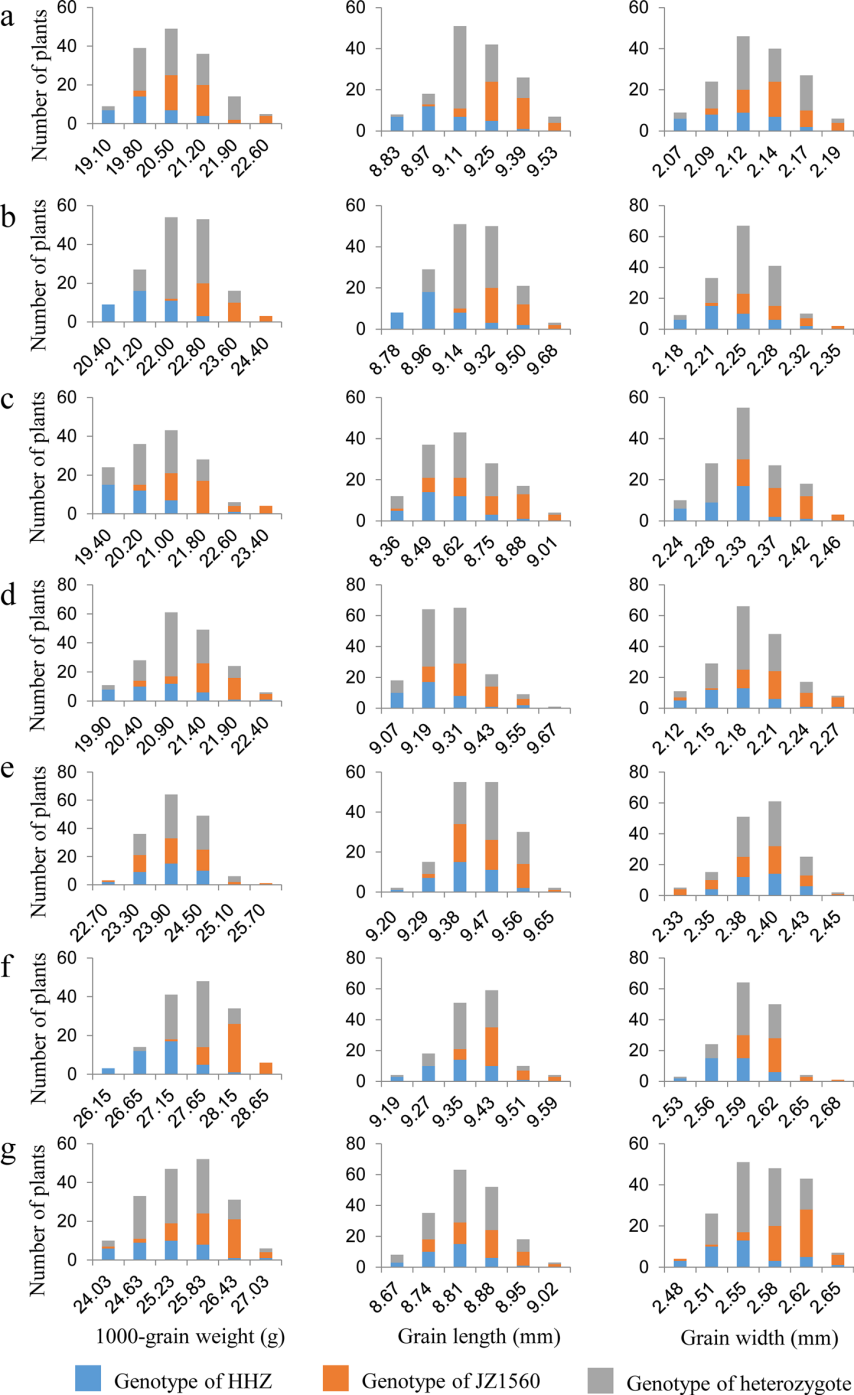
Distributions of 1000-grain weight, grain length, and grain width in the three BC_3_F_3_ and four BC_3_F_4_ populations. (**a**) YD1-1. (**b**) YD1-2. (**c**) YD1-3. (**d**) YD2-1. (**e**) YD2-2. (**f**) YD2-3. (**g**) YD2-4.

For further validation and dissection of *qTGW1-2*, we conducted another experiment using advanced backcross populations with the genetic background of HHZ. Three BC_3_F_3_ and four BC_3_F_4_ populations with the sequential segregating regions covering the interval JD1009-RM6840 were established and planted in three rice-growing seasons (Table 2; Fig. 1). For each plant of the seven populations, TGW, GL and GW were measured, and the distribution tendencies of the three traits are shown in Fig. 3. TGW, GL and GW showed continuous distributions in all the populations, but differentiation between the HHZ and JZ1560 homozygous genotypes was also observed. Except for in YD2-2 population, concentrations of the HHZ and JZ1560 homozygous plants were obviously distributed in the low- and high-value areas for TGW, GL and GW in YD1-1, YD1-2, YD1-3, YD2-1, YD2-3 and YD2-4 populations, which suggested *qTGW1-2* was segregated in these populations (Fig. 3). Similar frequency distribution of the three traits of the HHZ and JZ1560 homozygous plants was observed in YD2-2 population.

**Figure 4:**
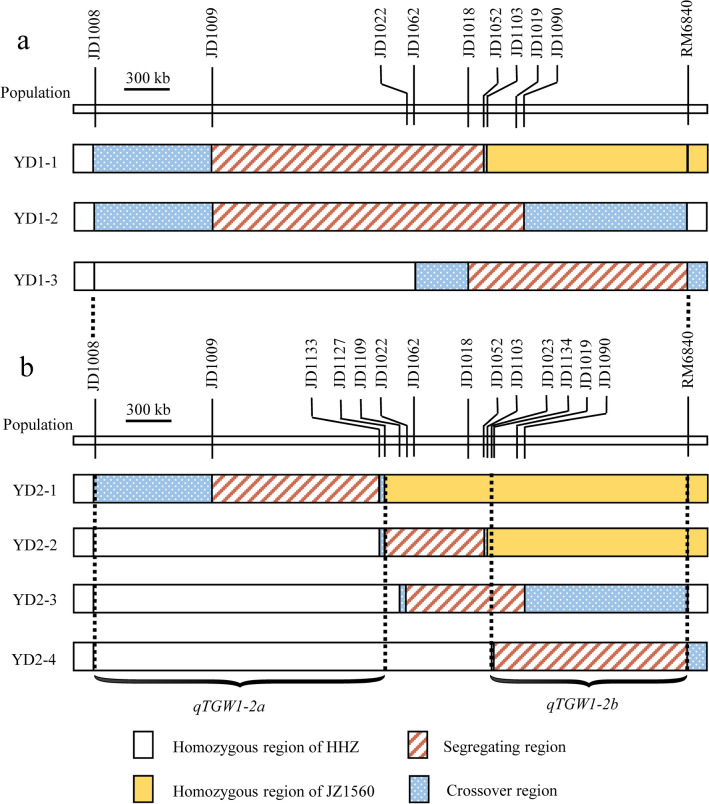
Genotypic compositions of the three BC_3_F_3_ and four BC_3_F_4_ populations in the segregating regions.

Combined the genotype and phenotype information of each plant in the seven populations, seven segmental linkage maps were constructed (Fig. 4). Results of QTL analysis for TGW, GL and GW using YD1-1, YD1-2 and YD1-3 populations were presented in Table 4. Significant QTL effects for the three traits were observed in all the three BC_3_F_3_ populations. For TGW, GL and GW, the enhancing alleles were derived from JZ1560 in all the three populations. In YD1-1, the additive effects were 0.54 g for TGW, 0.15 mm for GL and 0.02 mm for GW, explaining 22.8%, 38.6% and 16.9% of the phenotypic variances, respectively. In YD1-2, the additive effects were 0.87 g for TGW, 0.18 mm for GL and 0.02 mm for GW, explaining 45.6%, 39.6% and 13.6% of the phenotypic variations, respectively. In YD1-3, the additive effects were 0.72 g for TGW, 0.08 mm for GL and 0.03 mm for GW, explaining 31.4%, 13.4% and 22.7% of the phenotypic variances, respectively. The segregating region of YD1-2 overlapped the segregating regions of YD1-1 and YD1-3, and larger additive effects for TGW and GL were detected in YD1-2 than that in YD1-1 and YD1-3 populations (Fig. 4a). The results suggested there might be more than one QTL in the whole segregating region, which is consistent with the results of QTL analysis in the three F_9_ RH-derived populations.

**Table 4:** QTL detected for TGW, GL, and GW in seven backcross populations in BC_3_F_3_ or BC_3_F_4._ TGW, 1000-grain weight (g); GL, grain length (mm); GW, grain width (mm); *A*, additive effect of replacing a HHZ allele with a JZ1560 allele; *D*, dominance effect; *R*^*2*^, proportion of phenotypic variance explained by the QTL effect; ns, no significance.

Population	Segregating region	Trait	*LOD*	*A*	*D*	*R*^2^ (%)
YD1-1	JD1009-JD1052	TGW	9.84	0.54	0.15	22.8
		GL	18.55	0.15	0.02	38.6
		GW	7.08	0.02	0.01	16.9
YD1-2	JD1009-JD1090	TGW	25.03	0.87	0.07	45.6
		GL	20.80	0.18	0.01	39.6
		GW	6.01	0.02	0.00	13.6
YD1-3	JD1018-RM6840	TGW	15.13	0.72	− 0.09	31.4
		GL	5.81	0.08	0.00	13.4
		GW	9.82	0.03	− 0.01	22.7
YD2-1	JD1009-JD1133	TGW	10.82	0.40	− 0.06	24.2
		GL	6.37	0.07	− 0.01	15.0
		GW	7.44	0.02	− 0.01	12.3
YD2-2	JD1127-JD1052	TGW	ns	ns	ns	ns
		GL	ns	ns	ns	ns
		GW	ns	ns	ns	ns
YD2-3	JD1022-JD1090	TGW	24.66	0.52	− 0.05	51.8
		GL	11.23	0.06	− 0.01	28.0
		GW	11.20	0.02	0.00	27.9
YD2-4	JD1134-RM6840	TGW	9.90	0.47	− 0.15	22.0
		GL	4.24	0.04	0.00	10.1
		GW	9.85	0.03	− 0.01	21.7

For further delimitation of *qTGW1-2*, we conducted QTL analysis of four BC_3_F_4_ populations with sequential segregating regions covering the interval JD1009-RM6840, which were developed from four recombinant plants in the YD1-1, YD1-2 and YD1-3 populations (Table 4). Significant QTL effects were detected in YD2-1, YD2-3 and YD2-4 but not in YD2-2. In YD2-1, the additive effects were 0.40 g for TGW, 0.07 mm for GL and 0.02 mm for GW, explaining 24.2%, 15.0% and 12.3% of the phenotypic variances, respectively. In view of non-significant QTL effects detected in YD2-2, the YD2-1 population was segregated for *qTGW1-2a* only. Thus, for the region of *qTGW1-2a*, the segregating region of YD2-2 was excluded and the cross-over region on the left of the InDel marker JD1127 should be included. In YD2-3 and YD2-4, significant QTL effects were identified for all the three traits. The enhancing alleles were all derived from JZ1560 with the additive effects of 0.52 g and 0.47 g for TGW, 0.06 mm and 0.04 mm for GL, 0.02 mm and 0.03 mm for GW in YD2-3 and YD2-4 populations, respectively. Considering that the similar additive effects of the three traits were detected in YD2-3 and YD2-4, we confirmed that the QTL *qTGW1-2b* should be located in the common segregating region and two cross-over regions flanked by JD1023 and RM6840 (Fig. 4b), which is in corresponding to the results of QTL analysis in the seven F_10_ RH-derived populations.

### Fine-mapping of *qTGW1-2b* using four newly developed NIL-F_2_ populations

**Figure 5:**
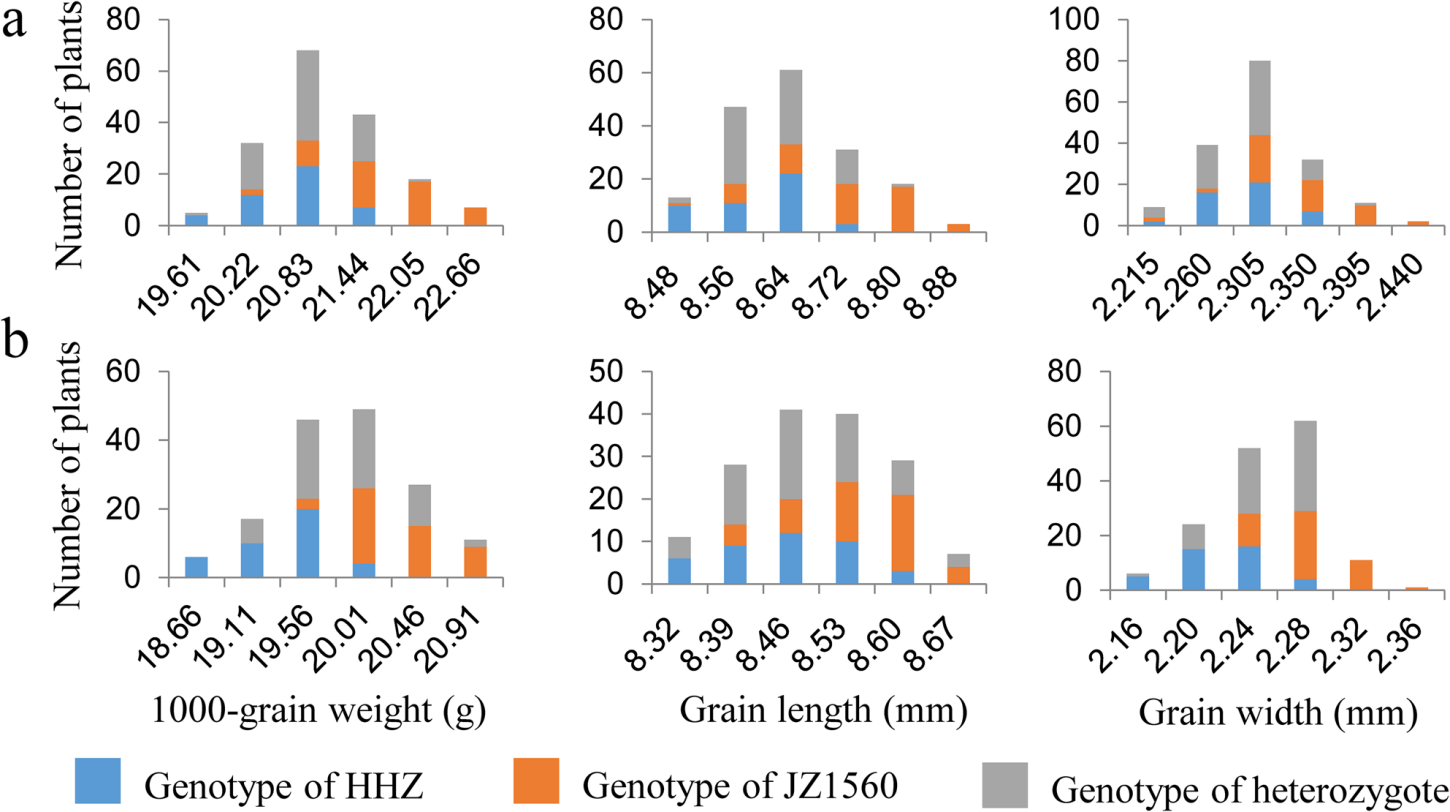
Distributions of 1000-grain weight, grain length, and grain width in the two BC_3_F_5_ populations. (**a**) YD3-1. (**b**) YD3-2.

For further delimitation of qTGW1*-2b*, two RH-derived populations in F_11_ and two backcross populations in BC_3_F_5_ with sequential segregating regions covering the interval JD1117-RM6840 were developed from the last generation (Table 2; Fig. 1). Descriptive statistics of TGW, GL and GW in the two RH-derived populations in F_11_ are shown in Table 5. Three traits showed the same continuous distribution with low skewness and kurtosis as the previous RH-derived populations. Similarly, the distribution tendencies of the three traits are descripted in Fig. 5. The plants with HHZ and JZ1560 homozygous genotypes concentrated to low- and high-value areas, which indicated that *qTGW1-2b* was segregated in the two BC_3_F_5_ populations.

**Table 5:** Phenotypic performance of TGW, GL and GW in two RH-derived populations in F_11._ TGW, 1000-grain weight (g); GL, grain length (mm); GW, grain width (mm).

Population	Trait	Mean	SD	CV	Range	Skew	Kurt
YC3-1	TGW	32.48	1.64	0.05	28.41–36.15	− 0.16	− 0.20
	GL	10.89	0.17	0.02	10.46–11.36	0.28	− 0.32
	GW	3.28	0.07	0.02	3.13–3.45	0.22	− 0.23
YC3-2	TGW	33.44	1.56	0.05	28.81–36.80	− 0.26	− 0.30
	GL	10.78	0.12	0.01	10.49–11.21	0.13	0.83
	GW	3.28	0.06	0.02	3.06–3.41	− 0.61	0.53

**Figure 6:**
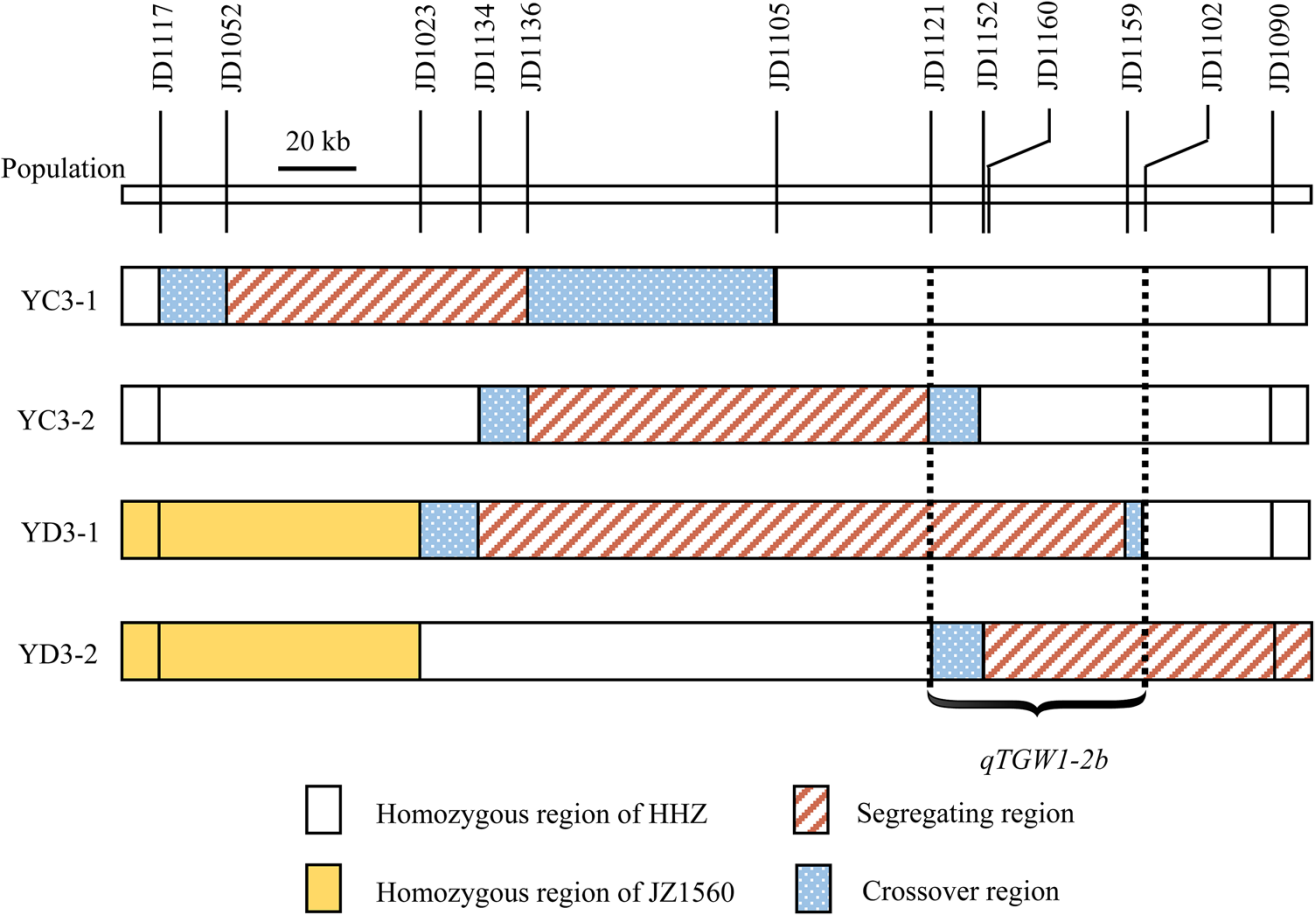
Genotypic compositions of the two F_11_ and two BC_3_F_5_ populations in the segregating regions.

Genotypic compositions of the two F_11_ and two BC_3_F_5_ populations in the segregating regions are shown in Fig. 6. The segregating region covered the whole original interval of *qTGW1-2b*. QTL analysis results for TGW, GL and GW using these populations are shown in Table 6. Significant QTL effects were detected in YD3-1 and YD3-2 but not in YC3-1 and YC3-2. In YD3-1, the additive effects were 0.46 g for TGW, 0.06 mm for GL and 0.02 mm for GW, explaining 36.7%, 29.1% and 20.3% of the phenotypic variances, respectively. In YD3-2, the additive effects for TGW, GL and GW were 0.46 g, 0.05 mm and 0.03 mm, explaining 44.1%, 16.3% and 30.6% of phenotypic variations, respectively. Similar additive effects with the enhancing alleles derived from JZ1560 in the same direction comfirmed the existence of *qTGW1-2b*. In view of non-significant QTL effects detected in YC3-1 and YC3-2, *qTGW1-2b* was delimited to the common segregating region of YD3-1 and YD3-2 flanked by JD1121 and JD1102 which corresponded to a 54.8-kb region in the Nipponbare genome.

**Table 6:** QTLs detected for TGW, GL, and GW in two RH-derived populations in F_11_ and two backcross populations in BC_3_F_5._ TGW, 1000-grain weight (g); GL, grain length (mm); GW, grain width (mm); *A*, additive effect of replacing a HHZ allele with a JZ1560 allele; *D*, dominance effect; *R*^2^, proportion of phenotypic variance explained by the QTL effect; ns, no significance.

Population	Segregating region	Trait	*LOD*	*A*	*D*	*R*^2^ (%)
YC3-1	JD1052-JD1136	TGW	ns	ns	ns	ns
		GL	ns	ns	ns	ns
		GW	ns	ns	ns	ns
YC3-2	JD1136-JD1121	TGW	ns	ns	ns	ns
		GL	ns	ns	ns	ns
		GW	ns	ns	ns	ns
YD3-1	JD1134-JD1159	TGW	17.30	0.46	− 0.33	36.7
		GL	13.02	0.06	− 0.02	29.1
		GW	8.59	0.02	− 0.02	20.3
YD3-2	JD1152-RM6840	TGW	21.42	0.46	0.01	44.1
		GL	6.78	0.05	− 0.02	16.3
		GW	15.34	0.03	0.00	30.6

### Candidate gene analysis of *qTGW1-2b*

According to Rice Genome Annotation Project (http://rice.plantbiology.msu.edu), there are nine candidate genes in the 54.8-kb region for *qTGW1-2b*. Gene ID and products are listed in Table 7. Based on 100× whole genome re-sequencing of HHZ and JZ1560^23^, we found five genes showing polymorphic sites between the two parents in the target 54.8-kb region. Among them, two genes showed 3-bp or 5-bp deletion in the UTR of the genes whereas one gene showed a 5-bp deletion in the intron. Only two genes showed polymorphic sites in the coding region between the two parents. *LOC_Os01g72480* showed a 9-bp insertion in the first exon in JZ1560, which encodes a C3HC4-type RING finger E3 ubiquitin ligase of the RING/U-box superfamily, whereas *LOC_Os01g72500* encoding a retrotransposon protein showed a 3-bp deletion in the first exon in JZ1560. According to the 9-bp sequence difference in *LOC_Os01g72480*, one InDel marker JD1160 was designed to genotype YC3-1, YC3-2, YD3-1 and YD3-2 segregating populations. As shown in Fig. 6, genotypes of YD3-1 and YD3-2 that showed significant additive effects were heterozygous at the polymorphic site of JD1160. In the segregating populations, the JZ1560 homozygous plants with 9-bp insertion in the first exon of *LOC_Os01g72480* were obviously distributed in the high-value areas for all the three grain traits, whereas the HHZ homozygous plants were distributed in the low-value areas (Fig. 5), indicating that the 9-bp insertion might result in the phenotypic viriations of TGW, GL and GW.

**Table 7:** Candidate genes in the target region of *qTGW1-2b*.

Candidate gene	Gene product	Polymorphic information
*LOC_Os01g72440*	Hypothetical protein	None
*LOC_Os01g72450*	DNA binding protein	None
*LOC_Os01g72460*	NADPH quinone oxidoreductase	None
*LOC_Os01g72470*	Expressed protein	3-bp deletion in 3’UTR
*LOC_Os01g72480*	C3HC4-type RING finger E3 ubiquitin ligase	9-bp insertion in the first exon
*LOC_Os01g72490*	LRP1	5-bp deletion in 5’UTR
*LOC_Os01g72500*	Retrotransposon protein	3-bp deletion in the first exon
*LOC_Os01g72510*	Eukaryotic aspartyl protease domain containing protein	None
*LOC_Os01g72520*	Phosphoesterase family protein	5-bp deletion in the first intron

## Discussion

In the past decade, significant progress has been achieved in the isolation and functional characterization of QTLs for yield traits in rice, especially for grain size and weight^30,31,35^. To date, more than 500 QTLs for rice grain weight distributing throughout the 12 chromosomes (http://www.gramene.org) have been identified in the primary mapping. Only 20 of them with major effects for grain size and weight have been cloned, which distributed on the eight chromosomes except for chromosomes 1, 10, 11 and 12. Majority of QTLs controlling grain size and weight show minor effects and are difficult to be cloned. In the present study, one minor QTL for grain weight and GL, *qTGW1-2*, was validated and dissected. No QTL for grain size and weight has been validated or cloned in the target region of *qTGW1-2*.

*qTGW1-2* is a stably expressed QTL with minor effects for TGW, GL and GW. In this study, the minor effects of *qTGW1-2* on TGW, GL and GW were stably observed across different populations, generations, rice-growing seasons and locations. Although the values of additive effects appeared to be changed in different experiments, the enhancing allele was derived from JZ1560 and the direction of additive effects remained unchanged for all the three grain traits. *qTGW1-2* was further dissected into *qTGW1-2a* and *qTGW1-2b*. Due to the clustering distribution of QTLs controlling grain size and weight, genetic dissection of QTL regions into different QTLs has been frequently reported^36–40^. Based on the results of QTL analysis using seven RH-derived populations in F_10_ and four BC_3_F_4_ populations, *qTGW1-2* was dissected into two QTLs (*qTGW1-2a* and *qTGW1-2b*), which were responsible for all the three grain traits. Compared the target intervals of these two QTLs in F_10_ RH-derived populations and BC_3_F_4_ populations, *qTGW1-2a* was delimited to the marker interval flanked by JD1139 and JD1127 (~ 978.2-kb), and *qTGW1-2b* was narrowed down into a ~ 186.0 kb region flanked by JD1023 and JD1102. Subsequently, two RH-derived populations in F_11_ and two BC_3_F_5_ populations were constructed, and *qTGW1-2b* was successfully fine-mapped to an accurate region flanked by JD1121 and JD1102, corresponding to a 54.8-kb region in the Nipponbare genome.

According to 100 × whole genome re-sequencing of HHZ and JZ1560, only two genes showed insertion/deletion polymorphic sites in the exons. *LOC_Os01g72480* encoding a C3HC4-type RING finger E3 ubiquitin ligase of the RING/U-box superfamily, showed a 9-bp insertion in the first exon of JZ1560 compared to that of HHZ. It was reported that ubiquitin pathway is one of the most important regulatory pathways for seed development^35^. *LOC_Os01g72500* showed a 3-bp deletion in the first exon and encodes a retrotransposon protein. No related evidence was reported that *LOC_Os01g72500* involves in seed development. Therefore, *LOC_Os01g72480* might be the possible candidate gene to regulate grain size and weight.

NIL-F_2_ populations developed from RHs of the RIL population and advanced backcross individuals are ideal materials for identifying minor QTLs. In this study, these two types of populations were both constructed to validate and dissect the minor QTL, *qTGW1-2*. In our previous primary mapping, *qTGW1-2* was detected to be a pleiotropic QTL for TGW and GL because *qTGW1-2* for TGW and *qGL1-2* for GL were mapped in the same marker interval JD1009-JD1019 across two years in the HHZ/JZ1560 RIL population. No significant QTL effect for GW was observed in the target interval of *qTGW1-2*. Compared with the QTL effects of *qTGW1-2* in the primary mapping RIL population, *qTGW1-2* showed small but significant additive effects on GW besides TGW and GL in the NIL-F_2_ populations derived from both RHs and advanced backcross individuals. Furthermore, *qTGW1-2* explained much higher phenotypic variation in NIL-F_2_ populations. For TGW, *qTGW1-2* explained 9.20%-10.72% of phenotypic variations in the HHZ/JZ1560 RIL population. In the present study, *qTGW1-2* accounted for 17.0%-23.8% and 22.8%-31.4% of the total phenotypic variations in RH-derived F_9_ populations and BC_3_F_3_ populations, respectively. These results indicated that elimination of genetic background noise in the NIL-F_2_ population increased the detection power of minor QTLs.

Compared to the cloned QTLs, both *qTGW1-2a* and *qTGW1-2b* showed relatively smaller effects, which might increase the difficulty in map-based cloning of them. The most likely candidate gene of *qTGW1-2b*, *LOC_Os01g72480* encoding an E3 ubiquitin ligase might control grain size and weight through the ubiquitin-proteasome pathway. In rice, *GW2* and *WTG1* encoding an E3 ubiquitin ligase and a deubiquitinase respectively also regulate grain size by ubiquitin regulatory pathway^9,41^. In this study, we successfully delimited *qTGW1-2a* and *qTGW1-2b* to the marker intervals of JD1139-JD1127 (~ 978.2-kb) and JD1121-JD1102 (~ 54.8-kb) at the end of chromosome 1, respectively. The closely linked InDel markers, such as JD1160, JD1140 and JD1102, could be used in the marker assistant selection of *qTGW1-2a* and *qTGW1-2b* to improve rice varieties. Compared with the major QTLs, the breeders are hard to select the varieties which carry minor QTLs by the phenotypic identification. However, benefited from the development of gene markers or tightly linked markers, molecular marker assistant selection of the minor QTLs can be performed with high accuracy and efficiency in the early stage of breeding.

## Materials and methods

### Development of F_9_, F_10_ or F_11_ RH-derived NIL-F_2_ populations

Based on the previous genotyping of the HHZ/JZ1560 RIL population with 208 DNA markers^23^, one RIL plant with the residual heterozygous region covering JD1009-RM6840 was identified and selfed to produce one F_8_ population. To validate the genetic effect of *qTGW1-2*, three recombinant plants with sequential heterozygous regions covering JD1009-RM6840 were selected and selfed to construct three RH-derived F_2_ populations in F_9_ named as YC1-1, YC1-2 and YC1-3 (Fig. 1). These populations contained 120, 128 and 224 individuals, respectively. To further narrow down the marker interval of *qTGW1-2*, seven recombinant plants with sequential heterozygous regions covering JD1009-RM6840 were further selected from the three F_9_ NIL-F_2_ populations and selfed to generate YC2-1, YC2-2, YC2-3, YC2-4, YC2-5, YC2-6 and YC2-7 RH-derived NIL-F_2_ populations in F_10_ (Fig. 1). For further delimitation of *qTGW1-2b*, two F_11_ NIL-F_2_ populations with sequential heterozygous segments covering JD1117-JD1152 named as YC3-1 and YC3-2 were constructed (Fig. 1). Except for the YC2-3 population with 104 individuals, each of the remaining six populations contained 190 individuals.

### Construction of NIL-F_2_ populations by consecutive backcrossing with the recurrent parent HHZ

The F_1_ plants derived from the cross between HHZ and JZ1560 were backcrossed with the recurrent parent HHZ for three consecutive generations. After screened by 208 DNA markers throughout the whole genome^23^, one BC_3_F_1_ (HHZ////HHZ///HHZ//HHZ/JZ1560) plant with the heterozygous region covering JD1009-RM6840 was selected and selfed to generate one BC_3_F_2_ population. To validate the genetic effect of *qTGW1-2*, three recombinant plants were selected from the BC_3_F_2_ population to construct three BC_3_F_3_ populations named as YD1-1, YD1-2 and YD1-3 (Fig. 1). These populations carried sequential heterozygous regions covering JD1009-RM6840. To further dissect *qTGW1-2*, four BC_3_F_3_ recombinant plants with sequential heterozygous regions covering JD1009-RM6840 were selected and selfed to produce YD2-1, YD2-2, YD2-3 and YD2-4 BC_3_F_4_ populations with sequential heterozygous regions covering JD1009-RM6840 (Fig. 1). Furthermore, two BC_3_F_5_ NIL-F_2_ populations named as YD3-1 and YD3-2 with sequential heterozygous segments covering JD1023-RM6840 were developed to narrow down the target region of *qTGW1-2b* (Fig. 1). Each of the populations mentioned above contained 190 individuals.

### Field trails and phenotypic evaluation

The twenty-one populations were planted in the experimental stations of the China National Rice Research Institute, which were located at Hangzhou (120.2° E, 30.3° N) in Zhejiang Province or Lingshui (110.0° E, 18.5° N) in Hainan Province, China (Table 2). All the populations were planted with 16.7 cm between plants and 26.7 cm between rows. Field management followed local agricultural practice. At maturity, plants were harvested individually and sun-dried. Approximately 200 fully filled grains were selected by 3.5 mol/L NaCl solution. TGW, GL and GW were measured by the methods of Zhang et al^32^.

### DNA marker analysis

DNA of each plant in the populations was extracted from the tender leaves following the method of Zheng et al^42^. PCR amplification was performed according to Chen et al^43^. The PCR profile was as follows: pre-denaturation of 3 min at 94°C, 30 cycles of 30 s at 94°C, 30 s at 55°C and 45 s at 72°C, followed by a final incubation at 72°C for 8 min. For genotyping the populations, a total of 23 insertion and deletion markers were designed with Primer3.0 (http://primer3.ut.ee/) according to the sequence differences between HHZ and JZ1560 detected by 100× whole genome re-sequencing. Primer sequences, physical position and PCR product sizes of HHZ and JZ1560 are listed in Table 8.

**Table 8:** InDel markers developed in our study.

Name	Forward primer (5′–3′)	Reverse primer (5′–3′)	Physical position (bp)	Product size (bp)	
				HHZ	JZ1560
JD1018	ATCGGTGCTGAGTGCTGAC	GAGGTGATGCGATTGGGAC	41,727,488–41,727,915	417	427
JD1022	CCTGGAAACGGAGCGTATTT	GCAGTCGGTGGTGTAGTGGA	41,318,246–41,318,669	403	423
JD1023	GAAATGTGAGCGTCAGAAGT	GTCGTCCGTTATGTTCAAGTA	41,879,696–41,880,129	413	433
JD1049	AATGCAAACGTGAGAAATTG	AGGTAGAGAAAAACAGGCGA	41,749,752–41,749,987	211	235
JD1052	TGAATTGGGTCATATCCTTGT	ATTGCTGGAATCGTATCGTAG	41,830,564–41,830,867	283	303
JD1062	TGGAGAGTAAACAGAAAAGC	GGTGACAAGTAAGAAACGAG	41,372,303–41,372,597	284	294
JD1088	AAACATGAATCGTGAAAGCA	ATCGGATCAACCACAGTAGC	40,966,716–40,966,939	243	223
JD1090	GATGGATTGATGATAGCGCA	AACTCGTACAACCCAAGTGG	42,098,975–42,099,272	277	297
JD1102	GTGATGCTCCTTTTCAATG	AGAATCGGGATACCACCT	42,065,654–42,065,834	169	180
JD1103	TCCTTTCACCAATCACGG	AAGGGTTGGGAAGAGGCT	41,850,660–41,850,861	182	201
JD1105	ATTCTGAATATCTGGTTGGATC	GGGGTTGACTTTGGAAAA	41,971,742–41,971,957	225	215
JD1113	GCTTCGGTTTATTAGGGC	GGTCACTGGTCAGGGTCA	41,109,254–41,109,638	349	376
JD1117	TGCCCATTGCTATGTAGA	CAACCTCACTGCTTACGG	41,812,497–41,812,749	221	252
JD1121	CCAGTCACCGAAGGAAGT	CAGAGCAGATGAGCAGGA	42,010,780–42,011,269	522	489
JD1127	GTAGCGTGCCTCCTGTTT	GGTCCAACTCTGGTTTCTTC	41,180,095–41,173,703	155	168
JD1133	ACCTGATATTATTCGGGACA	GCAGCAACTTCAACTTCACT	41,139,023–41,139,367	351	364
JD1134	GGCGTATGCTTATTGGAT	AAATAGACTTTTCTCACCCT	41,894,706–41,895,072	385	366
JD1136	ATTAAGCATACATGAAGCC	AAGCCAACTATCACAAACTA	41,907,772–41,908,134	339	362
JD1139	ACATTTTGTCCGCTACTG	CCACAACCATTCTTTCGT	40,195,295–40,195,588	327	293
JD1140	GAAAATGGGTGCTCAAAA	GCTTACTAAAACGGCAGAA	40,363,254–40,363,645	423	391
JD1152	CATAACTCGCCTGGAAAC	TCAACTTAGACCCCGTTT	42,024,093–40,324,345	252	232
JD1159	ATGCTTCAAGTTACTCCCT	ACTCTTCCGCCTAATCTC	42,060,810–42,061,277	486	497
JD1160	CGAACCCCTCGCCTCCTT	CGTGGTCGCATCCCTTGA	42,025,727–42,026,161	425	434

### Data analysis

Genetic maps of each population were constructed using Mapmaker/Exp 3.0, in which genetic distances between markers were presented in centiMorgans (cM) derived with Kosambi function. QTL analysis was performed with Windows QTL Cartographer 2.5, and the LOD value of 3.0 was taken as the threshold value.

### Permission statement

Experimental research and field studies on plants, including the collection of plant materials, comply with relevant institutional, national, and international guidelines and legislation.

## Conclusions

A total of 21 NIL-F_2_ populations were constructed to validate and dissect a minor and stably expressed QTL, *qTGW1-2*. Based on the QTL analysis of these genetic populations, *qTGW1-2* was successfully validated to control grain length, width and weight with the enhancing allele derived from JZ1560. Furthermore, *qTGW1-2* was further dissected into two QTLs, *qTGW1-2a* and *qTGW1-2b*, which were respectively narrowed down to the marker intervals of JD1139-JD1127 (~978.2-kb) and JD1121-JD1102 (~54.8-kb). These results supplied important basis for further map-based cloning and molecular design breeding in rice.
